# Do Future Teachers Believe that Video Games Help Learning?

**DOI:** 10.1007/s10758-021-09586-3

**Published:** 2021-12-16

**Authors:** Beatriz Cabellos, Daniel L. Sánchez, Juan-Ignacio Pozo

**Affiliations:** Department of Basic Psychology, Faculty of Psychology, Autonomous University of Madrid, C/ Ivan Pavlov, 6, 28049 Madrid, Spain

**Keywords:** Learning, Video games, Teacher training, Beliefs, Questionnaire, University education

## Abstract

One of the factors associated with the educational use of video games is the conception that teachers and students have about their educative usefulness. However, there are no studies that identify what aspects are considered more effective to learn with video games and what kind of learning is more accessible using them. This study aims at identifying pre-service teachers’ conceptions regarding video game use for learning and specifically to know what aspects and learning they consider are more feasible. Likewise, we analyzed the pedagogical training effect of these conceptions for three groups of university students: primary pre-service teachers (who received general pedagogical training), secondary pre-service teachers (who received pedagogical training in only one area of knowledge) and other university students without pedagogical training. We applied a questionnaire to a sample of 422 university students. This questionnaire had two dimensions that differentiated between the pragmatic and epistemic uses of video games for learning and three dimensions about the different verbal, procedural and attitudinal learning which can be achieved with them. The results showed wide acceptance of video games as a learning resource in university students, but in particular secondary pre-service teachers pointed out higher possibilities of achieving learning with video games than primary pre-service teachers. On the other hand, university students pointed out more learning when video games were used in an epistemic way. In addition, they considered video games favor more verbal and procedural learnings than attitudinal ones. In conclusion, despite the positive conceptions of the students about learning with video games, we observed a less positive pattern in pre-service teachers with general pedagogical training. These results suggest that video game incorporation in schools is not being carried out fruitfully by education faculties. Therefore, we advocated for 21st-century training that optimized new conceptions and uses of video games.

## Introduction and State of the Art

Video games (VG) are currently one of the mass entertainment media with the greatest reach in the youth population and perhaps the most economically important cultural industry. Young people devote a large part of their time to this use, which explains why the implications they have on the cognitive, affective, and social development of children and young people, as well as their possible educational uses, have recently come to the fore.

Research carried out in recent decades shows that the VG, in general, has a moderate positive effect in different areas of the curriculum (Boyle et al., 2016; Mayer, 2019). However, it also shows disparate results, when not limited, in these learnings, especially in the case of complex conceptual learning (Boyle et al., 2016; Clark et al., 2016; Mayer, 2019; Merchant et al., 2014). These types of learning, factual and commonly taught in school (See Pozo et al., 2021), have been prioritized over other procedural or attitudinal learning. In the case of procedural learning, some research indicates the benefits of VG in attention (Bediou et al., 2018; Parong et al., 2017), spatial cognition (Bediou et al., 2018; Boot et al., 2008), or speed in processing (Dye et al., 2009). In attitudinal learning, the results are controversial. Some authors (Anderson et al., 2010; Bavelier et al., 2011; Greenfield, 2014) highlight that VG favor aggressive behaviors and a lack of compliance with social commitments. However, other research (Greitemeyer & Mügge, 2014; Passmore & Holder, 2014) indicates improvement in prosocial attitudes or other types of positive behaviors, as in the study by Peng (2009) in which it was found that healthy eating behaviors were favored through a VG.

On the other hand, various studies also show that better learning is achieved when the VG use is mediated or scaffolded by the teaching action (de Aldama & Pozo, 2020; Clark et al., 2016; Mayer, 2019). Therefore, a critical variable of the effectiveness of VG as learning resources would be the use that teachers or educational mediators make of them in instructional contexts (de Aldama & Pozo, 2020; Gros & Garrido, 2008; Hérbert & Jenson, 2017). In turn, various studies have shown those teacher beliefs (Buehl & Beck, 2015) are a variable that allows predicting in part the use that teachers will make of a technological resource (Ermert et al., 2012; Tondeur et al., 2017) and specifically of the VG (Bourgonjon et al., 2010; de Grove et al., 2012).

We think that the educational effectiveness of VG may depend not only on their design but also on how teachers conceive their use and what expectations they have about the learning they can produce. In this sense, this study is aimed at analyzing the teacher beliefs about educational VG use.

Therefore, we are interested in knowing if these beliefs fit the data that the research has shown on the effectiveness of VG for learning, with consideration of what type of results are promoted with the educational VG use and if these results are due to the VG characteristics (Kors, et al., 2015; Mitgustsch & Alvarado, 2012; Smith & Just, 2017) or if learning needs to be guided through instructional interventions or external scaffoldings in their use (de Aldama & Pozo, 2020; Hébert & Jenson, 2017).

Regarding this last point about what type of VG use can be more effective, it can be explained by the different ways of playing or using these VG. Traditionally, it has been assumed that VG favor learning due to specific characteristics linked to their own design (Kors, et al., 2015; Mitgustsch & Alvarado, 2012; Smith & Just, 2017) such as the immediate feedback they provide to the player's actions, their playful character that favors motivation, by stimulating the dopaminergic reward system with its reinforcers (Aprea & Ifenthaler, 2021; Eseryer et al., 2014; Greenfield, 2014; Howard-Jones et al., 2011; Kim & Ifenthaler, 2019), the facility to embody oneself in the characters, which favors the cognitive or emotional empathy (Alhabash & Wise, 2012; Aprea & Ifenthaler, 2021; Bachen et al., 2016), or the enactive learning they promote, from the action itself and without the need for reflection on the actions (Pozo, 2017).

Resorting to Kirsh and Maglio's (1994) distinction between the pragmatic -success-oriented- and epistemic -knowledge-oriented- functions of cognitive activity, from this perspective, learning through VG would be based on the pragmatic use of them. The activity itself and the feedback received would promote associative, implicit, and embodied learning (Pozo, 2017; de Aldama, 2020) without the need to reflect on what has been learned.

However, this pragmatic orientation would also explain some limitations of learning based on VG: the scarce conceptual learning or the change of deeply rooted procedures or behaviors that cannot be modified only through implicit associative learning. These learnings require setting not only pragmatic goals, but also epistemic ones which promote reflection and an explicit relation of these actions with other previous learning. From this other perspective, the importance of favoring explicit learning through additional scaffolding to guide these epistemic goals is highlighted (Clark et al., 2016; Mayer, 2019). The aim would be to favor reflection, deliberate exploration, or explicitness of learners' implicit theories to rewrite them (Pozo, 2017; Barzilai & Blau, 2014; Karmiloff-Smith, 1992). Several studies show that deeper learning is achieved when the VG is oriented to epistemic goals favored by external scaffolding (de Aldama & Pozo, 2020; Barzilai & Blau, 2014).

### What Variables Influence the Conceptions About the Educational Use of Video Games?

Regrettably, despite the multiple educational possibilities of the VG and the importance of teachers' mediation to achieve learning with them, their use has been low (Ray et al., 2014). For instance, in a previous study (Pozo et al., under review), we analyzed the frequency with which Spanish teachers made use of VG in the classroom, finding that 68.18% of them had never used VG in class to promote learning. Paradoxically, this same study also identified that teachers had more positive conceptions of their educational possibilities the more they used them. Therefore, if we take into account that it is the youngest users who play with VG the most, we could expect their conceptions are more positive in terms of the educational possibilities they offer.

In this sense, some studies like in Bourgonjon et al. (2010) also identified that secondary school students who reported the most experience with VG were also the most likely to use them in class.

This frequency of VG use can be considered a critical variable regarding the teacher training in their use in educational contexts. However, there seems to be a common lack of training when using them in this context in different countries (Al Mulhim, 2014; Alqurashi & Alqurashi, 2017; Ertzberger, 2009; Hébert & Jenson, 2017; Ketelhut & Schifter, 2011; Ouahbi et al., 2016). Similarly, in Spain, where this study was carried out, teacher training regarding educational VG use, in general, is scarce, as evidenced over and over again in different studies (AEVI & GfK, 2012; Conde-Cortabitarte et al., 2020; Gros & Garrido, 2008; Martín del Pozo et al., 2017).

In the field of teacher training, the problem persists. For example, Barbour et al. (2009) again pointed out the difficulty of pre-service students when they use VG as an educational resource. It is noteworthy that this problem does not seem to be exclusive to the VG use but can also be extrapolated to the ICT use as an educational resource. For example, Luo et al. (2017) identified a lack of ICT training in USA students. In the same way, the problem is again also reflected in Spain. For example, Herrada-Valverde and Herrada-Valverde (2011) identified that the treatment given to training in ICT in teaching degrees in Spain is diversiform and scarce, available to a few pre-service teachers. This picture of the national panorama coincides with the opinions reported by pre-service teachers about the training they receive in the faculties of education: students pointed out that their knowledge of ICT is insufficient, they were unaware of their didactic potential, and did not know how to use them in the classroom (Gutiérrez-Martín et al., 2010).

For all these reasons, we believe it is important to consider the frequency of VG use by pre-service teachers and its influence on conceptions to identify if there are differences to experienced teachers. We consider that future teachers, for generational reasons, are more exposed to these resources, and differences could therefore be reflected concerning the veterans. On the other hand, we are interested in identifying whether this lack of training, which is assumed from the research already described, affects future teachers' beliefs and expectations of VG use differently when they are compared with other university students, of the same age, without specific pedagogical training.

We have already pointed out the importance of the Frequency of VG Recreational use and its relationship with pedagogical training using VG in education. However, we must not forget that other factors also seem to affect the conceptions regarding VG usage as an educational resource. For example, Bourgonjon et al. (2010), with a secondary students’ sample noted a positive, linear relationship between intention to use them and the learning opportunities attributed to the resource. In the specific case of education degree students, the results are similar: pre-service teachers who use VG often report more positive attitudes towards them (Martín del Pozo et al., 2017). Such frequency of use also seems to influence samples of practicing teachers (de Grove et al., 2012; Noraddin & Kian, 2014). In the case of intention to use VG in class, there seems to be a positive relationship concerning VG attitudes in samples of teachers (Bourgonjon et al., 2013; de Grove et al., 2012).

Similarly, the influence of gender on beliefs about educational VG use has also been detected, with male trainee teachers reporting to be more in favor of using them in this way than females (Marín-Díaz et al., 2019; Martín del Pozo, et al., 2017). However, for in-service teachers the results are confusing. Whereas Alqurashi and Williams (2019) indicated women had better *attitudes* than men towards using VG, Noraddin and Kian (2014) could not find any *gender* differences.

It should be noted that a relevant variable in this field of study, but with contradictory results, is the educational stage at which VG are aimed. Studies such as that by Hsu et al. (2017) have shown that teachers of elementary grades are more optimistic about the possibilities of VG as an educational resource than those of higher ones. However, other studies have found no differences (Alqurashi & Williams, 2019; Pozo et al., under review). In the case of Spain, these differences may be due not only to the difference in the content taught at the various stages but also to the different training that primary and secondary school teachers receive. While teachers for children up to the age of 12 are trained in pedagogy and didactics over four years, secondary school teachers are only trained for this for a few months and only after they have specialized in a particular area of knowledge. Therefore, this factor could be expected to influence students' conceptions of VG use as an educational resource because there are significant differences in the pedagogical training received.

Considering the aspects reviewed in this introduction, this paper proposes to identify what conceptions both primary and secondary pre-service teachers have about educative VG use (which as we have seen differ in the amount of pedagogical training received), in addition to identifying these conceptions in the rest of university students, regardless of their educational level. Likewise, we will also take into account the relationship of several variables that the aforementioned studies highlight in terms of these conceptions. To this end, we have the following objectives:

1. To find out the beliefs about whether VG favor learning in educational contexts and how pedagogical training, gender, frequency of VG recreational use, and behavioral intention relate to these beliefs.
2. To identify which goals of VG (pragmatic and epistemic) enhance learning best and how pedagogical training is related to these. Also, how gender, frequency of VG recreational use, and behavioral intention impact these outcomes.
3. To identify the beliefs about the outcomes VG can achieve (verbal, procedural, or attitudinal learning) and how pedagogical training is related to these. Also, how gender, frequency of VG recreational use, and behavioral intention impact these outcomes.

## Materials and Method

### Task and Procedure

In this study, we used the questionnaire on conceptions which was used in our previous study with teachers (Pozo et al., under review). This questionnaire had the approval of the ethics committee of the Autonomous University of Madrid. The questionnaire comprised three sections. The first part included an informed consent of participation for the students. The second part required that the participants’personal and professional information be filled in (gender, pedagogical training, frequency of VG recreational use, and behavioral intention). The third part comprised 57 items, formulated both positively and negatively, regarding participants’ agreement towards certain statements on VG and learning on a 6-point Likert scale (1, strongly disagree; 2, quite disagree; 3, slightly disagree, 4, slightly agree; 5, quite disagree; 6, strongly agree) with a similar design to the test used by Bourgonjon (2010).

We grouped the 57 items into 5 dimensions divided into 15 subdimensions, as shown in Table 1. These dimensions were distributed between two scales: the first scale referred to VG characteristics and processes activated by its use which promote either a pragmatic or an epistemic approach to it. The pragmatic dimension refers to those characteristics of VG which are achieved by associative processes that the VG initiates. On the other hand, the epistemic dimension refers to those processes that favor autoregulation and reflection and that can occur both through play and external scaffolding. The second one was compounded by the three types of learning that VG use can favor: verbal(factual and conceptual learning), procedural(skills and processes), or attitudinal(social behavior). In Table 1, we also show an example for each item.

**Table 1:** Questionnaire dimensions on VG and learning

Dimensions	Subdimensions		Item examples
Pragmatic learning	Physical involvement		By having to perform physical actions to play a VG, learning is improved
	Emotional involvement		Getting accustomed to the character’s point of view restrains learning
	Interactivity		Seeing immediate consequences when playing a VG improves learning
	Goal motivation		Setting additional goals different from those stated in the VG restrains learning
Episthemic learning	Personalization		Being able to solve problems at the player’s pace improves learning
	Challenge		By facing tasks in which the player frequently fails, learning is restrained
	Teacher supervisión		When teachers provide additional knowledge concerning subjects present in the VG, learning is improved
	Prior knowledge		Having to apply any kind of prior knowledge when playing a VG restrains learning
	Metacognitive control		Planning, supervising, and consciously adjusting which actions are performed when playing a VG improves learning
Outcomes	Verbal learning	Data learning	It is difficult to learn multiplication tables from a VG
		Conceptual learning	Practicing different contents in a VG helps to understand hard to grasp concepts
	Procedural learning	Attentional learning	The amount of information that VG show produces attentional issues in daily life
		Transfer	VG favor applying their contents and concepts to analogous daily life situations
	Attitudinal learning	Integration and participation	Playing VG makes a person less sociable in everyday life
		Attitudes of tolerance and respect	VG help to assimilate values of tolerance towards different groups and individuals

### Participants

The questionnaire was sent telematically to all the Spanish faculties of education that included the email directory of their teaching staff on their website. We asked professors to distribute the questionnaire to their students. To promote student participation, they entered a raffle for € 75 in teaching material that was provided at the end of data collection. The sample was collected from February 8th, 2020, to March 30th, 2020, before the school lockdowns as a consequence of the COVID-19 pandemic. We received 467 responses from students. From this sample, we eliminated 25 participants who took less than 5 min to respond or showed unreliable response patterns. The final sample comprised 442 participants aged 18–62 years (M = 23, SD = 5.62): 301 participants were primary pre-service teachers who were specifically training towards the teaching profession, 94 participants were secondary pre-service teachers trained in a specific subject area taking a course that qualified them to become secondary school teachers, and the remaining 32 participants were students at university taking degrees unrelated to education. Table 2 shows the personal information collected from the participants.

**Table 2:** Personal and professional information from participants

Variables	Categories	N	%
Gender	Men	97	22.15
	Women	341	77.85
	Others^a^	4	–
Age	30 years old or less	401	90.70
	More than 30 year sold	41	9.30
Pedagogical training	Primary pre-service teachers (General training in education)^b^	301	70.49
	Secondary pre-service teachers (Specific training in an area)	94	22.01
	Other university students without any training in education	32	7.49
	Others	15	–
Frequency of VG Recreational use (in their daily life)	Never	191	43.21
	Several days a month	139	31.59
	Several days a week or higher	112	25.45
Behavioral intention	No intention to use VG in classroom	238	53.85
	Maybe will use VG in classroom	136	30.77
	Definitely will use VG in classroom	68	15.38
Total		442	100

^a^Values marked as ‘Others’ were not taken into account in the analyses due to their low frequency

^b^The Primary pre-service teachers' category included Early Childhood Education pre-service teachers due to the pedagogical training of these students do not differ for the purpose of the study

### Data Analysis

To ensure the reliability of the dimensions, we carried out a Cronbach's alpha analysis, getting values above 0.75 for the five dimensions of the questionnaire (pragmatic use factors, epistemic use factors, verbal outcomes, procedural outcomes, and attitudinal outcomes) and 0.96 for the total scale. As for the subdimensions, all scored above 0.65 except for motivation, challenge, prior knowledge, concept learning, and integration and participation, with reliabilities over 0.50.

To operationalize the analysis of the dependent variables of the questionnaire, we calculated the mean of the items for each subdimension and dimension separately. These points were treated as scalar or range variables. We treated the independent variables considered in the questionnaire as categorical variables, except for the frequency of VG recreational use and the behavioral intention to use it, which were analyzed as ordinal variables due to their specific characteristics. We also analyzed whether the pedagogical training variable co-varied with the behavioral intention to control it if appropriate. However, no effects were found on the covariate which would have led us to control for it.

For aim 1 we conducted one-factor ANOVAs in which post-hoc analyses were carried out to see the differences between stated beliefs according to the different demographic variables included in Table 2. Tukey's correction was applied in these post-hoc analyses. We also conducted a regression analysis that identified what percentage of change in beliefs was explained by the variables involved. For objectives 2 and 3 we conducted repeated-measures one-factor ANOVAs identifying differences between dimensions and subdimensions. For this analysis, we added a second completely randomized factor comprising the demographic variables and analyzed the interaction effect and the differences provided by the post-hoc analyses using the Bonferroni correction. When *p* ≤ . 05 we consider the differences statistically significant. We performed all statistical analyses using SPSS version 26.

## Analysis and Results

### Do Students Believe Video Games can Promote Learning?

Considering the total sample of university students, independently of their pedagogical training, students have a positive belief about the possibilities of learning with VG (M = 4.42, SD = 0.61). These high scores can be observed in the most of subscales (see Table 3). Likewise, there are some differences among groups as shown below.

**Table 3:** Dimension means in each group

Variables	Categories	Mean (Standard Deviation)					
		Total beliefs	Pragmatic uses beliefs	Epistemic uses beliefs	Verbal learning beliefs	Procedural learning beliefs	Attitudinal learning beliefs
Gender	Men	4.50 (0.61)	4.58 (0.68)	4.66 (0.60)	4.53 (0.73)	4.50 (0.75)	3.94 (0.69)
	Women	4.39 (0.61)	4.44 (0.65)	4.59 (0.63)	4.47 (0.71)	4.39 (0.75)	3.79 (0.76)
Age	30 years old or less	4.42 (0.60)	4.47 (0.65)	4.61 (0.62)	4.48 (0.71)	4.42 (0.74)	3.82 (0.74)
	More than 30 years old	4.40 (0.68)	4.44 (0.78)	4.60 (0.64)	4.48 (0.69)	4.41 (0.80)	3.80 (0.80)
Pedagogical training	Primary pre-service teachers	4.36 (0.61)	4.42 (0.65)	4.55 (0.62)	4.42 (0.71)	4.38 (0.73)	3.77 (0.72)
	Secondary pre-service teachers	4.54 (0.59)	4.60 (0.62)	4.74 (0.57)	4.64 (0.67)	4.51 (0.75)	3.93 (0.78)
	Other university students	4.54 (0.64)	4.54 (0.74)	4.79 (0.58)	459 (0.76)	4.50 (0.84)	3.77 (0.72)
Frequency of VG Recreational use	Never	4.21 (0.60)	4.27 (0.64)	4.45 (0.65)	4.27 (0.69)	4.16 (0.73)	4.42 (0.75)
	Several days a month	4.45 (0.58)	4.46 (0.63)	4.63 (0.60)	4.51 (0.66)	4.53 (0.68)	3.55 (0.69)
	Several days a week or higher	4.72 (0.54)	4.82 (0.57)	4.84 (0.53)	4.81 (0.66)	4.72 (0.70)	3.89 (0.72)
Behavioral intention	No intention to use VG in classroom	4.23 (0.58)	4.28 (0.63)	4.45 (0.60)	4.28 (0.71)	4.20 (0.74)	3.59 (0.70)
	Maybe will use VG in classroom	4.53 (0.54)	4.59 (0.60)	4.69 (0.60)	4.61 (0.61)	4.55 (0.62)	3.95 (0.64)
	Definitely will use VG in classroom	4.85 (0.56)	4.88 (0.64)	4.99 (0.53)	4.92 (0.61)	4.91 (0.69)	4.35 (0.74)
Total scale		4.42 (0.61)	4.42 (0.61)	4.47 (0.66)	4.61 (0.62)	4.48 (0.71)	4.42 ( 0.75)

There are significant differences regarding the pedagogical training as well as the frequency of VG recreational use and the behavioral intention to use them for teaching. The primary pre-service teachers were less in favor than the secondary pre-service teachers (*p* < 0.05). Besides, the higher the frequency and behavioral intention to VG use university students pointed out, the more positive beliefs about learning with VG they reported (F = 54.68, *p* < 0.001 and F = 63.78, *p* < 0.001 respectively). We observed no gender differences.

From these variables, we carried out a regression model to identify which factors affected beliefs finding that pedagogical training, behavioral intention, and the frequency of VG recreational use explained 21% of the dependent variable variance (F = 37.65, *p* < 0.001, R^2^ = 0.21). The behavioral intention (B = 0.29, t = 6.54, *p* < 0.001) and frequency of VG use (B = 2.69, t = 6.01, *p* < 0.001) were the variables which explained more, above pedagogical training (B = 0.10, t = 2.33, *p* < 0.05).

### Which Features of Video Games Favor Learning According to the Students?

We analyzed whether there were differences between the type of VG that students believed generated the most learning. The results were conclusive: University students pointed out VG generated greater learning when they were used with epistemic (M = 4.61, SD = 0.62) rather than pragmatic goals (M = 4.47, SD = 0.66), F = 82.81, *p* < 0.001, η_p2_ = 0.16. In other words, they thought that their educational utility increased when they were integrated into curricular activities rather than just for the sake of playing.

**Fig. 1:**
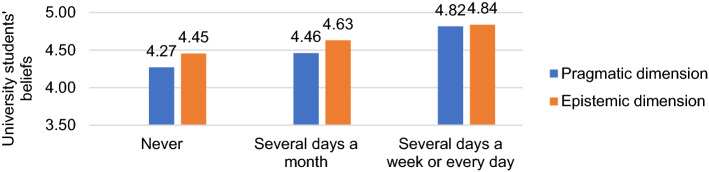
Effect of the interaction in the frequency of VG recreational use and pragmatic and epistemic dimensions

We were also interested in whether this preference for epistemic over pragmatic was influenced by the variables considered in the study. However, only significant differences were identified in the interaction effect on frequency of recreational use, F = 10.41, *p* < 0.001, η_p2_ = 0.05. Students who played daily with VG had closer attitudes between VG learning possibilities favored by both pragmatic and epistemic features than those who used them less (*p* < 0.001). In other words, students who played the most valued VG themselves as better educational resources than those who played the least. The increase in the pragmatic beliefs play is so notable that even the differences with epistemic play disappear. Nevertheless, the latter did also increase according to frequency of recreational use, but to a lesser extent (F = 52.59, *p* < 0.001 in pragmatic and F = 28.84, *p* < 0.001 in epistemic) (see Fig. 1).

**Fig. 2:**
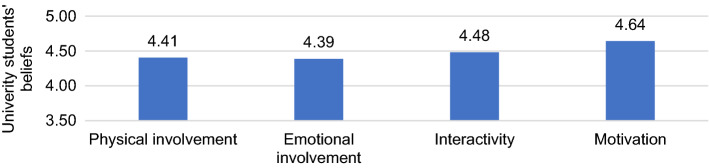
Means of the subdimensions in the pragmatic dimension

When taking these two dimensions separately, we only identified differences in epistemic factors in terms of pedagogical training, with secondary pre-service teachers being more positives than primary pre-service teachers (*p* < 0.05).We asked whether this disparity between the belief that VG can support learning because of their pragmatic and epistemic characteristics could be because of any subdimension. We identified differences both within the pragmatic subdimensions, F = 31.82, *p* < 0.001, η_p2_ = 0.05, and within the epistemic subdimensions, F = 38.55, *p* < 0.001, η_p2_ = 0.08. In the pragmatic ones (see Fig. 2), motivation was the most effective for learning, showing significant differences with all the other subdimensions (*p* < 0.001). Likewise, the characteristics of VG least valued for learning were those referring to bodily and emotional involvement, which in both cases also had significant differences with interactivity, which was considered slightly more useful for learning (*p* < 0.01).We also tried to identify whether the ratings towards each of the pragmatic goal-oriented VG characteristics were mediated by the variables analyzed. However, significant differences were only obtained when students were asked about the possibilities of emotional involvement to generate learning: secondary pre-service teachers valued the potential of emotional involvement in learning more highly than primary pre-service teachers (*p* < 0.001). Similarly, male students valued this dimension higher than female students (*p* < 0.01).

**Fig. 3:**
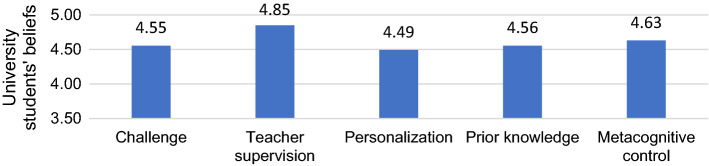
Means of the subdimensions in the epistemic dimension

For the epistemic subdimensions (see Fig. 3), students were more likely to positively value teacher supervision in using the VG for learning than the other dimensions (*p* < 0.001). However, differences were also identified between metacognitive management and the other three dimensions, which were considered less relevant to learning with VG (*p* < 0.05), and between personalization and prior knowledge, with the former being the least valued (*p* < 0.05). To sum up, the data suggest students consider that teacher mediation is necessary for VG to help learning, without ignoring the importance of activating the students' metacognitive processes.

As with the pragmatic subdimensions, we asked whether we could find differences among students for each of the epistemic characteristics associated with learning with VG. We only found differences regarding pedagogical training. Secondary pre-service teachers valued the prior knowledge to learn with VG more positively than primary pre-service teachers (*p* < 0.01).

### What do University Students Say they Learn Through VG?

In relation to the third aim, students believed VG favor mainly verbal (M = 4.48, SD = 0.71) and procedural learning (M = 4.42, SD = 0.75), relegating attitudinal learning to second place (M = 3.82, SD = 0.74) (F = 388.41, *p* < 0.001, η_p2_ = 0.47).

Subsequently, we analyzed whether these differences were modified by any of the demographic variables involved. We identified more positive beliefs to generate verbal learning in secondary pre-service teachers than primary pre-service teachers (*p* < 0.05). On the other hand, females considered VG would favor verbal learning more than procedural learning (*p* < 0.05), while we did not identify differences for the rest of the variables between these two types of outcomes.

**Fig. 4:**
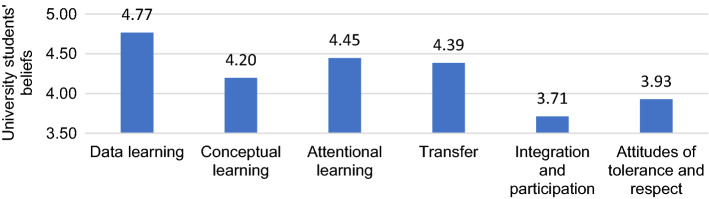
Means of the subdimensions for each type of learning outcomes

Finally, we analyzed whether there were differences between verbal, procedural, and attitudinal types of learning that VG enhance. We identified differences between verbal (F = 259.51, *p* < 0.001, η_p2_ = 0.37) and attitudinal learning (F = 36.73, *p* < . 001, η_p2_ = 0.08), with no differences between procedural learning. The students pointed out that in verbal learning, VG could mainly favor the learning of data instead of the learning of concepts, which requires elaborate connections between areas of information. These were by far the most likely learning to be achieved according to the students. However, students identified that VG would not be suitable for promoting attitudes of integration and participation when compared with attitudes of tolerance and respect. The latter, although also considered unlikely to be achieved with VG, did not show such a pessimistic tendency (see Fig. 4).

Finally, we asked whether the subtype of learning could be associated with any student characteristics. In this sense, we perceived differences for data learning, attentional learning, and fostering attitudes of tolerance and respect. In particular, students with teacher training in a specific area of knowledge pointed out more possibilities to learn data and attitudes of tolerance and respect while using VG than those with general teacher training (*p* < 0.05). Likewise, males believed the VG were more conducive to attentional learning than females (*p* < 0.01).

## Discussion and Conclusions

We will now return to the most relevant study findings to outline the considerations we consider appropriate. In general, our results show a positive assessment of students towards learning with VG, confirming the results of Salas-Rueda (2018) with a sample of university students as well as those of Hsu and Chiou (2011) and Ruggiero (2013) with education degree students. Likewise, these data are similar to those reported with samples of practicing teachers in compulsory education (Alqurashi & Williams, 2019; Bourgonjon et al., 2013; Hsu et al., 2017; Ruggiero, 2013), among which we highlight the study already carried out by this research team (Pozo et al., under review).

However, this study considers aspects of the research uncovered up to this point, offering novel results that propose new challenges that we will report below. One of the notable aspects is the identification of VG conceptions regarding pragmatic or epistemic uses, that until now, had only been contemplated in VG practice to identify the learning obtained (de Aldama & Pozo, 2020; Clark et al., 2016; Mayer, 2019).

In terms of which VG characteristics did students attribute greater learning possibilities to, we identified a greater predisposition towards games with epistemic goals in which there was an external mediation that favors orientation towards different learning objectives. These data correspond to those taken from the same questionnaire in a sample of teachers (Author, under revision). Likewise, in terms of differences in pedagogical training, secondary school teachers are more supportive of the use of epistemic objectives than primary school teachers, which paradoxically implies that they attach greater importance to the teaching role as a facilitator of learning with these resources.

On the other hand, university students who spend more time playing VG value the epistemic thinking features as much as the pragmatic aspects, which may be linked to a certain knowledge about VG that allows them to value potential learning aspects that are not detected by teachers whose use is more amateurish. These results confirm the findings of studies aimed at analyzing the characteristics of VG as learning enhancers (Alhabash & Wise, 2012; Aprea & Ifenthaler, 2021; Bachen et al., 2016; Eseryel, et al., 2014; Ge & Ifenthaler, 2017; Howard-Jones et al., 2011; Huizinga et al., 2017; Kim & Ifenthaler, 2019; Malinverni & Pares, 2014).

When we identified which specific factors the students in our research identified as important in teaching with VG, teacher supervision stood out. They consider that the role of teacher scaffolding activates certain processes that facilitate learning (Kors, et al., 2015; Mitgustsch & Alvarado, 2012; Smith & Just, 2017). These data were similar to those from the previous study with a sample of teachers and to those already established in the scientific literature, which highlights the importance of scaffolding to generate learning (Clark et al., 2016; Mayer, 2019) beyond the learning possibilities that VG themselves promote (de Aldama & Pozo, 2020; Barzilai & Blau, 2014).

Another novel implication of the study is the distinction of students' conceptions of what types of learning can be offered with VG. In this sense, the students highlighted that VG facilitated verbal learning, followed by procedural learning, in contrast to attitudinal learning, which is relegated to last place. These results coincide with the study we carried out with the sample of teachers (Pozo et al., under review). This result could be expected, considering the common practices with ICT in the classrooms (Pozo et al., 2021). Verbal learning is the most frequent in school and this can be extrapolated to the use of any resource. Likewise, regarding attitudinal learning, there is a common belief, which we pointed out at the beginning, that VG can favor aggressive or antisocial behavior (Anderson et al., 2010; Bavelier et al., 2011; Greenfield, 2014). However, in recent times, an opposing trend has emerged that identifies the potential of VG for pro-social learning, as evidenced by the work of Darvasi (2016), Granic et al. (2014), Greitemeyer and Osswald (2009), Greitemeyer et al. (2010), and Passmore and Holder (2014).

On the other hand, we have identified some variables that affect students' beliefs about the potential of VG in learning. The most influential ones are the frequency of VG recreational use and the behavioral intention. The more students play, the more favorable their beliefs are, highly similarly to the study by Bourgonjon et al. (2010) with secondary school students and also with students trained in education (Marín-Díaz et al., 2019) and teachers (Pozo et al., under review; Bourgonjon et al., 2013; de Grove et al., 2012; Noraddin & Kian, 2014; Sánchez-Mena et al., 2017, 2019).

However, when we compared our results with those obtained in the previous study (Author, under revision) that we carried out with a sample of teachers, we found that although our sample of students used VG more, their conceptions were not more positive than the in-service teachers.

This same trend was also identified in the intention to use. The higher the intention to use, the more positive the conceptions regarding VG usage, which is in line with studies conducted with in-service teachers (Bourgonjon et al., 2013; de Grove et al., 2012), and among them ours (Author, under revision).

In short, the university students in our study have a favorable attitude towards VG use in the school environment. This mainly varies according to the behavioral intention and frequency of VG use in the classroom.

However, the most controversial data of this study is the role played by pedagogical training. Until now, it had not been identified how the role of pedagogical training affected the use of VG, which is a relevant aspect for this research field. The pedagogical training does not seem to favor more positive beliefs when using VG. In our data, primary pre-service teachers, who received 4 years in educational training, were more skeptical about using VG than students training to be secondary school teachers, who only received 1 year training in education. These data contrast with the results obtained by Manessis (2013), who notes that education degree students in higher grades had more positive conceptions towards VG. This difference in the results may be due to methodological differences between this research and ours. This study, unlike ours, uses the degree year as its variable identifying differences between first-year and last-year students.

On the other hand, one limitation of our study is that the sample of university students who were not related to education was very small (n = 32). This caused a high variance within the category itself, which affected the fact that, despite obtaining similar means to secondary students about the use of VG, no significant differences were identified regarding the elementary school students who received educational training throughout their stay at the university.

This fact could even indicate that these people who are not going to dedicate themselves to teaching are the most optimistic when it comes to using VG for educational purposes. We would therefore propose studies contemplating larger samples of this type of students without pedagogical training to identify possible differences in their conceptions concerning students with knowledge in education. Furthermore, it would be interesting to replicate these studies to identify the training differences between countries in pre-service teachers, or even cross-country studies. Further qualitative studies would also be pertinent, to identify pre-service teachers' conceptions.

But returning to our results, the obtaining of less positive attitudes in students with pedagogical training compared to those who received training for 1 year could indicate that this pedagogical training only improves expectations about VG use when this training is partly supported by their use, which does not seem to be the case of our sample.

This is in line with the studies which point out the lack of training in teachers when they use VG for learning (Alqurashi & Alqurashi, 2017; Conde-Cortabitarte et al., 2020; Ertzberger, 2009; Hébert & Jenson, 2017; Ketelhut & Schifter, 2011). Therefore, from the results obtained, it can be deduced that, as in the case of in-service teachers, pre-service teachers have a lack pedagogical training when using VG (Barbour et al., 2009) and in general in ICT (as suggested by the studies by Herrada-Valverde & Herrada-Valverde, 2011; Luo et al., 2017).

From our perspective, this lack of training regarding the educational potential of the VG reduces expectations and consequently the use that teachers make of them. For example, Hsu et al. (2013) identified positive relationships between Game Pedagogical Knowledge and Learning Opportunities, which showed that specific training in Game Pedagogical Knowledge was crucial not only to promote the expertise of teachers and future teachers in its use but also to promote more favorable attitudes towards its educative possibilities (An & Cao, 2017; Kenny & McDaniel, 2011; Martín del Pozo et al., 2017; Sánchez-Mena et al., 2017). Studies such as An and Cao (2017), Hsu et al. (2013), or Kenny and McDaniel (2011) point out that training is key not only to improving the perception of competence in VG use but also to improving conceptions regarding the perceived learning possibilities.

In this sense, it is becoming increasingly necessary to train future teachers in VG use in the classroom. The aim should be that teachers will be able to achieve the greatest educational potential possible in VG usage. In this study, educational training does not increase the educational potential that this resource can offer in teaching; on the contrary, it reduces it. Therefore, it is necessary to rethink the training received to integrate the VG use as another resource that can favor the formation of competencies in students. Moreover, such use must go beyond isolated predominantly verbal learning as it has been commonly carried out: pre-service teachers must be trained in the use of such resources for the integration of the different types of verbal, procedural, and attitudinal learning, with the aim to assume the teaching of 21st-century competencies (Ertmer et al., 2015).

They need to be aware of the importance of their role as mediators of learning with this resource, oriented towards generating epistemic attitudes in their students that favor their learning. Thus, it is necessary to reinforce teacher training in new technologies adapted to education and review the adoption of those that, like VG, are not initially designed for teaching. The evidence we have regarding the specificities of VG as learning devices as well as their impact on the social, cultural, and developmental spheres of the youth population is ample and should make us rethink how we are incorporating them into the ecosystems of schools and the educational community.

## Declarations

### Data Availability

The datasets used and/or analyzed during the current study are available from the corresponding author on reasonable request.

### Human or Animals Rights

Research involves university students. All procedures performed in studies involving human participants were in accordance with the ethical standards of the institutional and/or national research committee and with the 1964 Helsinki Declaration and its later amendments or comparable ethical standards. The study was approved by the Ethical Committee of the Autonomous University of Madrid.
